# NUT expression in primary lung tumours

**DOI:** 10.1186/s13000-015-0395-9

**Published:** 2015-09-05

**Authors:** Marius Lund-Iversen, Krystyna Kotanska Grøholt, Åslaug Helland, Elin Borgen, Odd Terje Brustugun

**Affiliations:** Department of Pathology, Oslo University Hospital, Radiumhospitalet 0310, Oslo, Norway; Department of Oncology, Oslo University Hospital, Radiumhospitalet 0310, Oslo, Norway; Ullernchausseen 70, N-0310 Oslo, Norway

## Abstract

**Background:**

Nuclear protein in testis (NUT) midline carcinomas (NMC) are rare, highly aggressive epithelial neoplasms, characterised by protein expression of NUT-fusion proteins which reflects the genetic translocation between chromosome 15 and 19. NMC occurs mainly in midline structures, but there are reports regarding occurrence in structures outside the midline.

We investigated specimens from 519 surgically resected lung carcinomas and carcinoid tumours for the presence of NUT protein using immunohistochemistry. Normal testis and two previously confirmed NMCs served as positive controls.

**Findings:**

All 483 evaluable cases (278 adenocarcinomas, 140 squamous cell carcinomas, 30 large cell carcinomas, 7 small cell carcinomas, 10 undifferentiated carcinomas and 18 carcinoids) were completely negative for expression of NUT protein.

**Conclusion:**

NUT gene rearrangement does not seem to be relevant in primary pulmonary carcinomas or carcinoid tumours of the lung.

Nuclear protein in testis (NUT) midline carcinomas (NMC) are rare, highly aggressive epithelial neoplasms with a median survival of 6.7 months [1]. They were originally described in midline structures above the diaphragm in paediatric and adolescent age groups, but their occurrence in older age groups and in other anatomic locations are described [2–4]. Since the first reports there are now indications of higher prevalence of NMC in adults than first anticipated [1].

NMCs are poorly differentiated neoplasms with morphologic and immunophenotypic features of undifferentiated carcinoma and squamous cell carcinoma [5]. They are genetically defined by chromosomal rearrangements of the NUT gene on chromosome 15; in approximately 70 % the gene is fused to bromodomain-containing protein 4 (BRD4) on chromosome 19 resulting in t(15;19) translocation. The remaining cases harbour BRD3 or other rare or uncharacterised fusion partners [2, 6].

Only a few cases of NMC with putative origin in the lung have been reported [5, 7–11].

Due to the relatively new discovery of this entity and the presumed rarity of this disease, underrecognition is probable [12].

Patients with surgically treated lung carcinomas generally have prolonged survival compared to patients with inoperable disease, but preoperative biopsy interpretation and exact histopathological classification is frequently challenging due to scarce amount of tissue. Immunohistochemical markers as thyroid transcribing factor-1 (TTF-1), Napsin A, p63 and p40 are often helpful in classification, but these markers do not predict the biological or metastatic potential. Still, detailed histopathological and genetic subclassification is increasingly demanded in the quest for personalised therapy, but should be balanced against tissue economics and prioritisation of relevant analyses.

The occurrence of NUT positive cases among surgically treated lung cancers are to the best of our knowledge not known, but is putatively low, and large series describing NMC in the lung are lacking [13]. It has been suggested that all low-differentiated tumours devoid of glandular differentiation and of non-skin origin should be tested for the presence of NUT protein [12]. To explore the eventual occurrence of NUT expression, and the relevance of NUT immunohistochemical analysis in routine diagnostics, we examined a large cohort of surgically treated lung cancers for NUT expression by a monoclonal antibody in a tissue micro array (TMA) set.

## Materials and methods

Tumour tissue was obtained from a cohort of lung cancer patients in stage Ia-IV surgically resected at the Oslo University Hospital – Rikshospitalet during the period 2006 – 2013. Written informed consent was obtained from all patients and the project was approved by the regional ethics committee. Twenty-two tissue micro array (TMA) blocks were prepared from 519 surgical specimens from different intrathoracical locations. All major histological types were included, and all grades of differentiation were represented. One mm punch biopsies in triplicate were selected from representative tumour areas based on hematoxylin & eosin stained slides from the tumours. In addition the TMA set contained normal lung tissue, lymphoid tissue and metastatic tissue in lymph nodes. The morphological classifications were given in routine pathological reports based on the surgical specimen.

### Immunohistochemistry

Freshly cut 4 μm sections were immunostained on a Ventana Benchmark Ultra platform with a rabbit monoclonal antibody to NUT protein, the clone C52B1 from Cell Signaling Technology, product number 3625, at 1:200 dilution. Ventana/Roche CC1 pretreatment buffer (“standard”, ie 64 min) was used for antigen retrieval. Detection system was OptiView DAB IHC Detection Kit, product number 760–700, Ventana Medical Systems/Roche Diagnostics, used with OptiView HQ Universal Linker, thus constituting a highly sensitive 3 layer detection system. Control sections containing NUT positive and negative tissue (normal testis and tonsil respectively) were included on every TMA slide. As a supplementary control, sections from two previously confirmed NMC cases were prepared concomitantly. Staining of all slides was performed in one single run on the Ventana platform.

## Findings

**Fig. 1:**
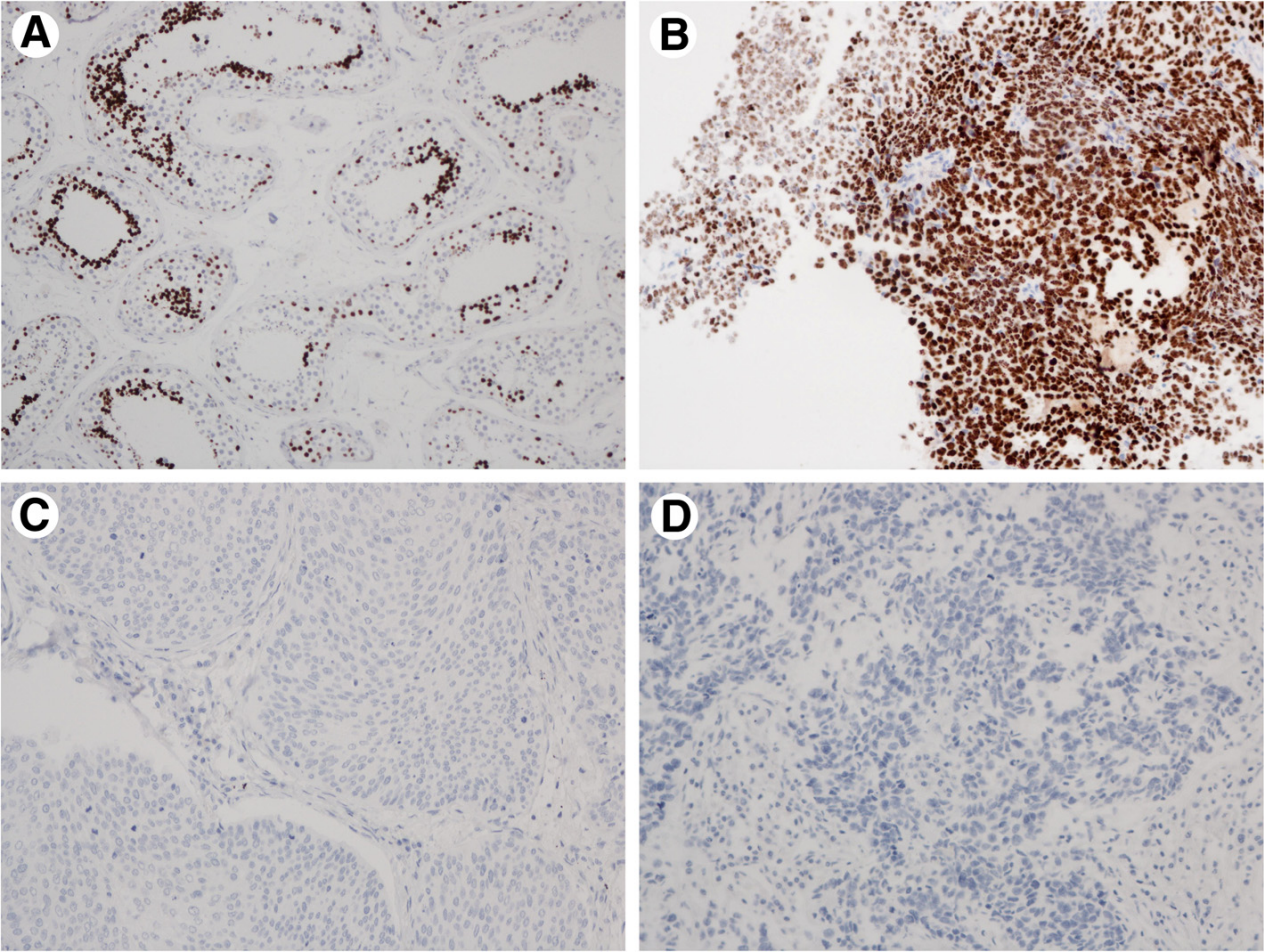
NUT-positive controls from testis (**a**) and one previously confirmed NUT midline carcinoma (**b**). NUT-negative lung carcinoma tissue (**c**, **d**)

**Fig. 2:**
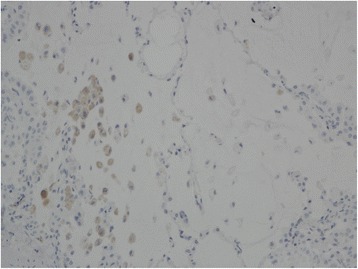
Unspecific cytoplasmic staining in alveolar macrophages

A total of 483 tumours were assessable for the anti NUT staining, the remaining samples were missing. 247 were from males and 236 from females, with median age 66.3 [range 33.9 - 87.0] years. Patient and tumour characteristics of the assessable cases are shown in Table 1. Approximately 20 % of the non-small cell carcinomas were poorly differentiated, and 10 % well differentiated (not shown). Every punch was evaluated by three consultant pathologists (MLI, KKG, EB). The previously confirmed NUT cases and the positive controls from testis had distinct nuclear staining without any background (Fig. 1a,b). All the tumour tissue, normal lung tissue and lymphoid tissue were completely negative for NUT staining (exemplified in Fig. 1c,d). Some alveolar macrophages had unspecific cytoplasmic staining (Fig. 2). None of the samples had equivocal staining.

**Table 1:** Characteristics of assessable tumours

Characteristic (*n* = 483)	Number	Percentage
Age (years) median, range	66.3 [33.9 – 87.0]	
Sex		
Female	236	48.9
Male	247	51.1
Smoking status		
Never	30	6.2
Previous	270	55.9
Current	183	37.9
Packyears (median)	28.5	
Histology		
Adenocarcinoma	278	57.6
Squamous cell carcinoma	140	29.0
Large cell carcinoma	30	6.2
Small cell carcinoma	7	1.4
Carcinoid	18	3.7
Carcinoma UNS	10	2.1
Stage		
I	276	57.1
II	135	28.0
III	68	14.0
IV	4	0.8
EGFR status		
Tested adenocarcinomas	240	
Mutated	27	11.3

## Discussion

There is an increased demand for accurate diagnosis and predictive tests in lung cancer.

Carcinomas harbouring NUT translocation are potentially treatable with selective bromodomain extra terminal (BET) inhibitors [14] which are available through ongoing phase I clinical trials (e.g. GSK525762 (NTC01587703) and TEN-010 (NCT01987362)). We have therefore investigated a large cohort of surgically resected lung cancers for the presence of NUT protein with immunohistochemistry. In 483 evaluable specimens of lung tumours from different intrathoracic locations we did not find any cases with NUT expression.

The sensitivity and specificity for the C52 B1 antibody is 87 and 100 % respectively among non-germ cell tumours [15], and the variance might be explained by different fusion partners to NUT. In our material none of the tumours had staining scored as faint or difficult to interpret. Approximately 20 % of the surgically resected non-small cell carcinomas in this material were poorly differentiated. These are the cases thought to be most likely NUT-positive, but neither of these stained positively in our material.

The golden standard for defining NMC is fluorescence in situ hybridization (FISH) with probes against NUT and any eventual fusion partner. The NUT-fusion protein product, investigated in this cohort, is highly predictive for the genetic translocation in tumour tissue [15]. The absence of use of FISH technique in this cohort marginally weakens the results, and we cannot exclude the possibility that a minority of the cases are false negative.

Although we studied a cohort of mostly early stage lung cancers, and we therefore cannot formally exclude the possibility that the cohort does not reflect more advanced, inoperable lung cancers, still almost 15 % of the cases were in stage III or IV. We therefore believe our findings to be universal regardless of resectability.

In conclusion, NUT-expressing primary carcinoma of the lung does not seem to be an underrecognised entity.

## Abbreviations

NUT: Nuclear protein in testis
NMC: Nuclear protein in testis (NUT) midline carcinomas
BRD: Bromodomain-containing protein
TTF-1: Thyroid transcribing factor-1
TMA: Tissue micro array
BET: Bromodomain extra terminal
FISH: Fluorescence in situ hybridization
MLI: Marius Lund-Iversen
KKG: Krystyna Kotanska Groholt
EB: Elin Borgen
